# Development of a 3D Collagen Model for the *In Vitro* Evaluation of Magnetic-assisted Osteogenesis

**DOI:** 10.1038/s41598-018-33455-2

**Published:** 2018-11-02

**Authors:** Zhiyu Yuan, Kaveh Memarzadeh, Abish S. Stephen, Robert P. Allaker, Robert A. Brown, Jie Huang

**Affiliations:** 1 Department of Mechanical Engineering, University College London, Torrington Place, London, WC1E 7JE UK; 2 Orthopaedic Research UK, Furlong House, 10A Chandos Street, London, W1G 9DQ UK; 3 Institute of Dentistry, Barts & The London School of Medicine and Dentistry, Queen Mary University of London, London, E1 2AT UK; 4 Tissue Repair and Engineering Centre, University College London, Stanmore Campus, London, HA7 4LP UK

## Abstract

Magnetic stimulation has been applied to bone regeneration, however, the cellular and molecular mechanisms of repair still require a better understanding. A three-dimensional (3D) collagen model was developed using plastic compression, which produces dense, cellular, mechanically strong native collagen structures. Osteoblast cells (MG-63) and magnetic iron oxide nanoparticles (IONPs) were incorporated into collagen gels to produce a range of cell-laden models. A magnetic bio-reactor to support cell growth under static magnetic fields (SMFs) was designed and fabricated by 3D printing. The influences of SMFs on cell proliferation, differentiation, extracellular matrix production, mineralisation and gene expression were evaluated. Polymerase chain reaction (PCR) further determined the effects of SMFs on the expression of runt-related transcription factor 2 (Runx2), osteonectin (ON), and bone morphogenic proteins 2 and 4 (BMP-2 and BMP-4). Results demonstrate that SMFs, IONPs and the collagen matrix can stimulate the proliferation, alkaline phosphatase production and mineralisation of MG-63 cells, by influencing matrix/cell interactions and encouraging the expression of Runx2, ON, BMP-2 and BMP-4. Therefore, the collagen model developed here not only offers a novel 3D bone model to better understand the effect of magnetic stimulation on osteogenesis, but also paves the way for further applications in tissue engineering and regenerative medicine.

## Introduction

Every year, approximately 850,000 patients suffer from bone fracture in the UK. The rate of non-union is suggested to be 5–10%, and the cost to the National Health Service (NHS) of treating non-union has been reported to range between £7,000 and £79,000 per person^1^, which has a substantial economic and quality of life impact. Bone regeneration is a physiologic process that replaces the injured bone with new bone thereby renewing the biological and mechanical properties of the injured site. It is a complicated metabolic process, which requires the interaction of many factors, including growth and differentiation factors, hormones, cytokines, and extracellular components. If these factors are inadequate or interrupted, healing will be delayed or impaired, resulting in non-union of the bone.

For more than a century, investigators have been developing alternative treatments that have aimed to resolve the bone fracture healing process, by physical or biological methods. The physical strategy includes the use of mechanical stimulation^2^, electrical stimulation^3^, electromagnetic stimulation^4^, and magnetic stimulation^5^. The biological approach involves the use of osteoconductive biomaterials^6^ and growth factors^7^. Wolff^8^ hypothesised that bone remodels in response to stress and strain, due to the fact that the structure of bone adapts to changes in its stress environment. This process is also known as mechanotransduction, which involves the conversion of a biophysical force into a biochemical response leading to changes in gene expression and cellular adaptation. Static magnetic fields (SMFs) have been applied to stimulate bone healing, possibly through the mechanism of mechanotransduction. It has been found that SMFs are capable of stimulating the osteogenesis of osteoblasts, by influencing their proliferation, differentiation, extracellular matrix production and mineralisation^9^. Despite the success of SMFs stimulations in several *in vivo* and *in vitro* studies, there remains two major concerns. Firstly, it is believed that bone responds to dynamic rather than static loading, and the stimulation is related to the peak strain magnitude and loading frequency^10^. Therefore in some of the cases, SMFs failed to demonstrate a positive effect on cell proliferation, differentiation and other factors. Secondly, for the situations where SMFs successfully stimulate the osteogenesis process, the molecular mechanisms of this phenomenon are not well understood.

*In vitro* models have been developed to study biological behaviour away from the intact living organism. Conventional *in vitro* models refer to the testing system where cell monolayers are cultured on a stiff or flat surface (two-dimensional), however, they differ from the natural tissue in many ways, such as structure, stiffness, cell/matrix interactions and attachments, and the concentration of essential nutrients^11^. It is evident that many cells respond differently when cultured in 3D compared to traditional 2D cultures, and often adopt more *in vivo*-like morphologies. Culturing cells in 3D radically alters the mechanical signals from those provided in 2D, thus affecting cell-receptor ligation, intercellular signalling and critical cell behaviours such as cellular migration. The 3D environment also influences the diffusion and adhesion of proteins, growth factors, and enzymes, which ensures cell viability and can influence function^12,13^. Maintaining cells in 3D systems, such as spheroids, micromass and pellets, promotes progression of osteoblastic differentiation leading to osteoblast cell maturation^14^. Therefore, it is necessary to develop a three-dimensional (3D) tissue/organ model to better mimic the natural environment for *in vitro* testing, which provides an important alternative to both complex *in vivo* whole organism approaches and 2D culture with its spatial limitations.

Collagen hydrogels have an established track record as potential 3D models. Collagen is the most abundant protein found in extracellular matrix (ECM), and provides desired properties such as an ECM-like scaffold, including water retention capacity, nano/micro-porosity to allow cells to grow and arrange in 3D, biodegradability, and pore inter-connectivity to allow free flow of oxygen and nutrients^15–17^. In addition, during tissue development and repair processes, collagen can interact directly with cells and influence several cellular activities, including adhesion, growth, differentiation, mineralisation of ECM, as well as the expression of growth factors and cytokines^15,18,19^. Due to these advantages, a large number of applications based on collagen hydrogels can be found, such as nerve guide tubes for peripheral nerves^20^, scaffolds for connective tissues^21^, 3D tumour models^11,22^, hematopoietic niche models^23^ to study the biological behaviours of various cells and genes^24–30^.

However, conventional collagen hydrogels are low in collagen density (approximately 0.2 to 0.5% of wet weight), with a large excess of fluid (>98%)^18^. This results in poor mechanical properties as well as the lack of orientated architecture. In order to construct a mechanically strong collagen based scaffold and maintain all useful properties, a novel approach known as plastic compression (PC) has been developed by Brown *et al*.^18^, with many studies having demonstrated the suitability of PC collagen scaffolds in tissue engineering applications, such as bone^19^, and cornea^31^. The PC process can be achieved by compressing conventional collagen gels, leading to the controlled expulsion of interstitial fluid. PC allows the fabrication of dense collagen constructs, which mimic the ECM fibrillary density, microstructure, and biological properties. With a single plastic compression system, collagen density in the resultant model can increase to 11–18%^18^. Furthermore, this construct has been tested to last for at least 5 weeks *in vivo*^32^. As regards 3D scaffolds, PC collagen hydrogels have been investigated to enhance the proliferation^33^, differentiation^19^ and mineralisation^19,34^ of osteoblasts. Recent studies also focused on the introduction of osteoconductive cues to further enhance the osteogenesis. Chitosan^35^ and bioactive glass particles^36^ have been incorporated into PC systems to enhance cell differentiation and mineralisation.

Osteoblastic cell lines have been used extensively for *in vitro* investigations, with the advantages of unlimited cell numbers, ease of culturing, reproducible growth characteristics and higher phenotypical stability as compared to primary cells^37^. Human osteosarcoma cell line (MG-63) have been used as an experimental model to study a variety of different osteoblastic functions such as adhesion, collagen synthesis, and osteocalcin production^38^. Recently, a number of studies have also successfully employed MG-63 cells as a model to investigate cell behaviour under magnetic fields^39,40^.

In this study, a novel multifunctional bio-mimetic 3D collagen model was developed and can be used as an *in vitro* platform to study the mechanisms of magnetic stimulation on osteogenesis. Both internal (iron oxide nanoparticles) and an external (SMFs) stimulus was introduced to the system to produce a range of cell-laden models. The collagen model was cultured in a magnetic bio-reactor for up to 42 days to evaluate several biological behaviours of osteoblasts, including proliferation, differentiation, mineralisation, gene expression and microstructure analysis.

## Results

### Microstructure of the cell-seeded collagen scaffolds

**Figure 1:**
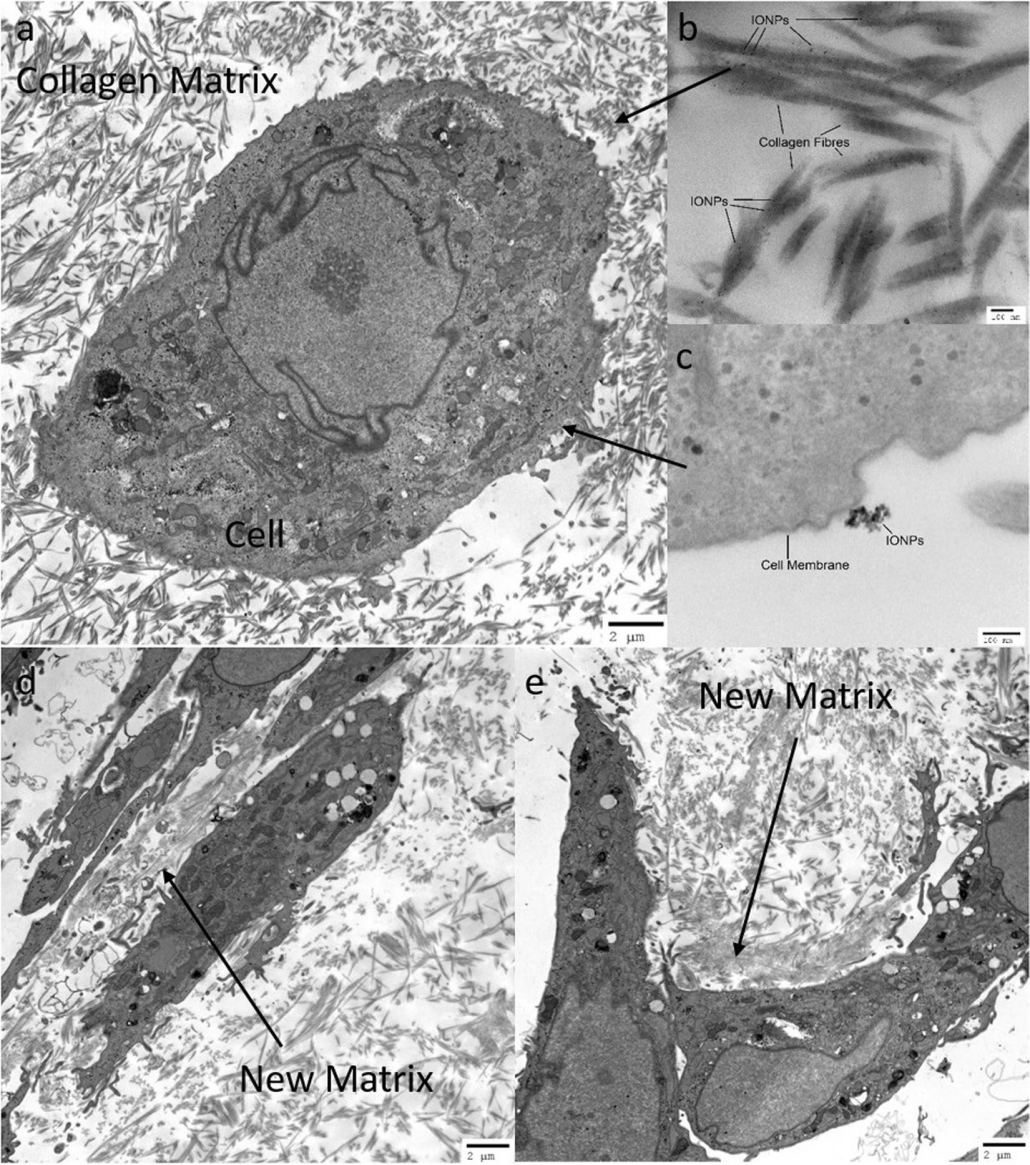
(**a**) Microstructure of cell loaded collagen scaffolds examined under TEM. Cells are surrounded by the collagen matrix, the collagen fibrils show no preferred direction around cells under control condition. IONPs can be identified (**b**) in the collagen matrix and (**c**) close to cell membranes. After 14 days, new matrix was synthesised by the cells (**d**) without SMFs and (**e**) with SMFs. Both conditions can lead to matrix synthesis after 14 days, with no significant difference.

The microstructure of the cell loaded collagen scaffolds was examined after 1, 7 and 14 days using TEM, with fibrous collagen structure and healthy attached cells shown (Fig. 1a). The incorporation of IONPs can be identified, as being found inside the cell in either agglomerated or separated state, close to the cell membrane or inside the collagen fibrils (Fig. 1b,c). As can be observed from Fig. 1d and e, new matrix was synthesised by the cells after 14 days (with and without the exposure to SMFs).

### Cell proliferation

**Figure 2:**
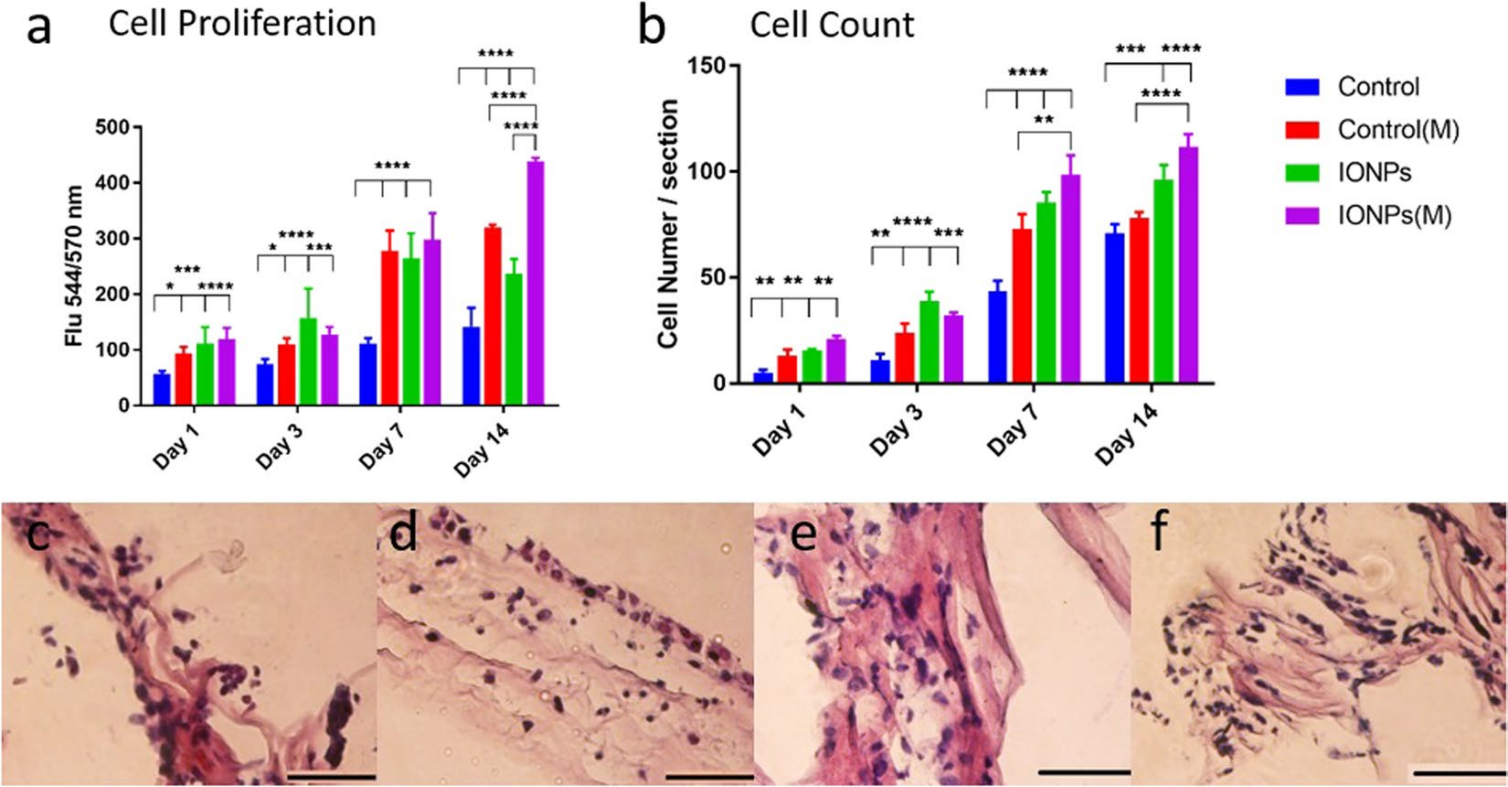
(**a**) A comparison of cell proliferation of MG-63 cells when cultured within collagen scaffolds with/without the incorporation of IONPs, with (M) and without the exposure of SMFs. Cell proliferation can be enhanced under SMFs with the incorporation of IONPs, indicating a stimulating effect. (**b**) A comparison of cell number of MG-63 cell lines from histology for IONPs with and without SMFs. The cell number represents the average from three sections of one sample. By incorporating additional IONPs, the cell proliferation can be further enhanced. The cellular responses of MG-63 cells when cultured in PC collagen scaffolds were also examined by histology, with no SMFs and no IONPs (**c**), SMFs (**d**), IONPs (**e**) and SMFs and IONPs (**f**). Scale bar = 100 *μ*m (n = 3, *p < 0.05, **p < 0.01, ***p < 0.001, ****p < 0.0001).

The proliferation of MG-63 cells was assessed by alamarBlue assay with results shown in Fig. 2a. From day 1, all treatments stimulated cell proliferation significantly, and this effect continued until day 14. At day 7, the cell proliferation with treatments almost doubled to that of the control, which indicates that all treatments had significant influences on cell proliferation, however, no differences between each treatment were observed. At day 14, significant differences between SMFs, IONPs and the combined on cell proliferation were observed. This demonstrates that employing SMFs or IONPs alone can only stimulate cell proliferation up to 7 days, whereas by combining both factors, the effect can be extended until 14 days. Cellular responses of compressed collagen gels seeded with MG-63 cell lines were obtained after 1, 3, 7 and 14 days of culture as shown in Fig. 2c–f. Histology was employed to examine the cellular responses. By visualising the functional cells inside the collagen scaffolds, the effects of SMFs and IONPs on cell proliferation examined by the AB assay can be validated. The cell numbers have been analysed quantitatively as shown in Fig. 2b. By employing SMFs alone, increased cell activity can be observed from 3 days but falls slightly after 14 days. This suggests that the SMFs alone can influence cell growth over short time periods but are not effective for longer durations. The incorporation of IONPs can further the stimulation effects for 14 days. This suggests that by incorporating IONPs, the effect of SMFs can be prolonged.

### Cell differentiation

**Figure 3:**
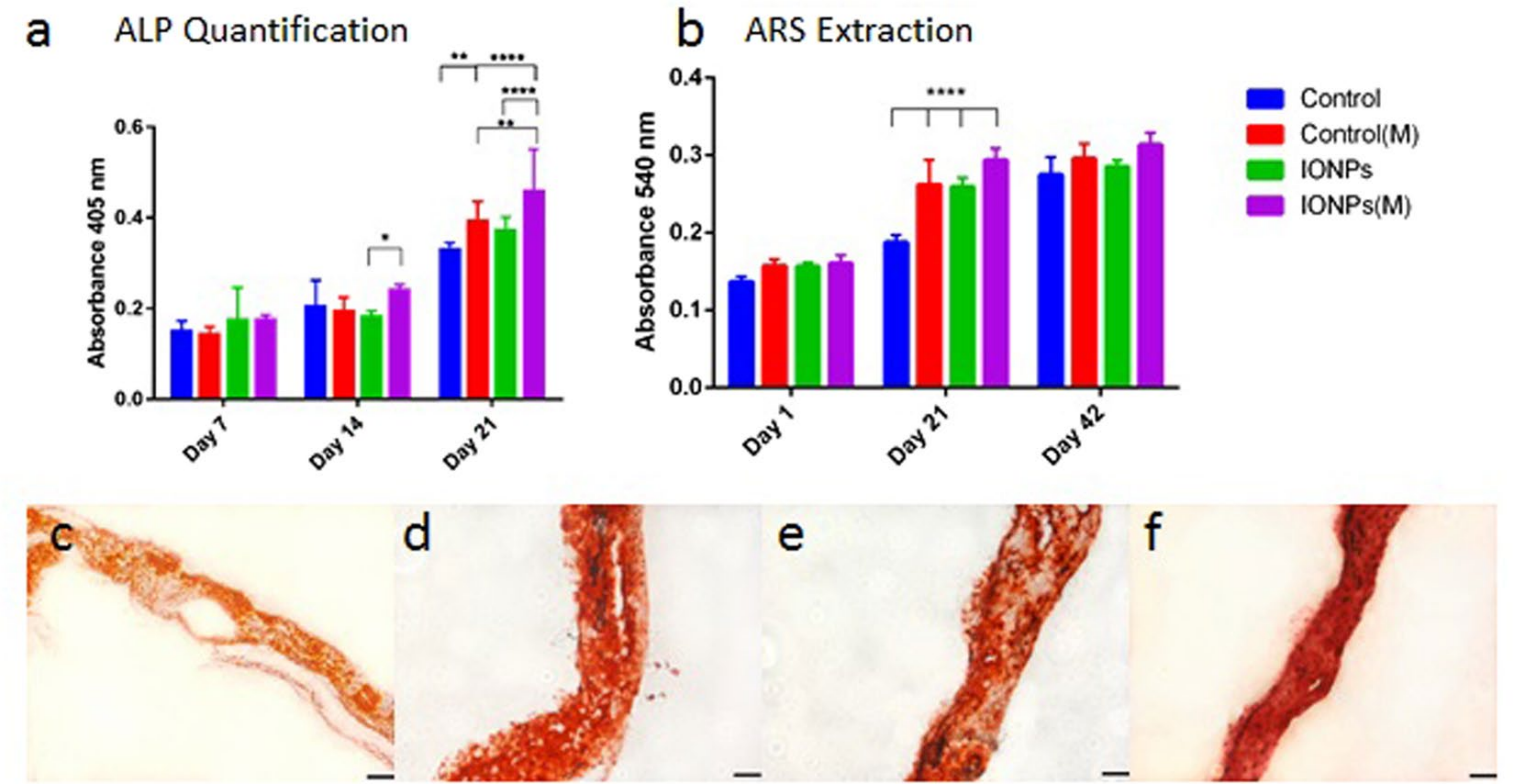
(**a**) A comparison of ALP production of MG-63 cells when cultured within collagen scaffolds with/without the incorporation of IONPs, with (M) and without the exposure of SMFs. (**b**) A comparison of cell mineralisation by extracting and quantifying ARS staining from scaffolds treated with and without SMFs exposure. Cell-loaded collagen scaffolds were incorporated with IONPs with a concentration of 100 *μ*g/ml, results were collected at 1, 21 and 42 days of culture time. (**c**) ARS of collagen scaffolds with the absence of SMFs and IONPs, (**d**) with SMFs, (**e**) with IONPs and (**f**) with SMFs and IONPs. Scale bar = 100 *μ*m (n = 3, *p < 0.05, **p < 0.01, ***p < 0.001, ****p < 0.0001).

Alkaline phosphatase (ALP) activities of collagen scaffolds with or without SMFs, and with/without the incorporation of IONPs were determined. As shown in Fig. 3a, no significant effect on ALP production was observed with SMFs, IONPs or the combination, up to 14 days. On exposure to SMFs for 21 days, the ALP production of the cells was up-regulated significantly under SMFs and the combination of SMFs and IONPs, whereas little difference was found with the incorporation of IONPs alone. When further combining the effect of IONPs and SMFs, the ALP production was significantly stimulated when compared to the SMFs (alone) and IONPs (alone) treatment.

### Cell mineralisation

ARS staining and quantification of mineralisation of cell-seeded collagen scaffolds are shown in Fig. 3. At day 1, mineralisation was not observed for all conditions. After 21 days, significant differences in the degree of mineralisation was identified, with a higher level for scaffolds treated with SMFs. After 42 days, all samples were fully stained by ARS, indicating complete mineralisation. Results indicated that SMFs can induce early mineralisation *in vitro*, with more significant simulation when IONPs were incorporated. However, after 42 days, extraction levels remained unchanged when compared to the control, indicating that the SMFs and IONPs were not able to promote mineralisation at this period.

### Gene expression: real-time polymerase chain reaction

**Figure 4:**
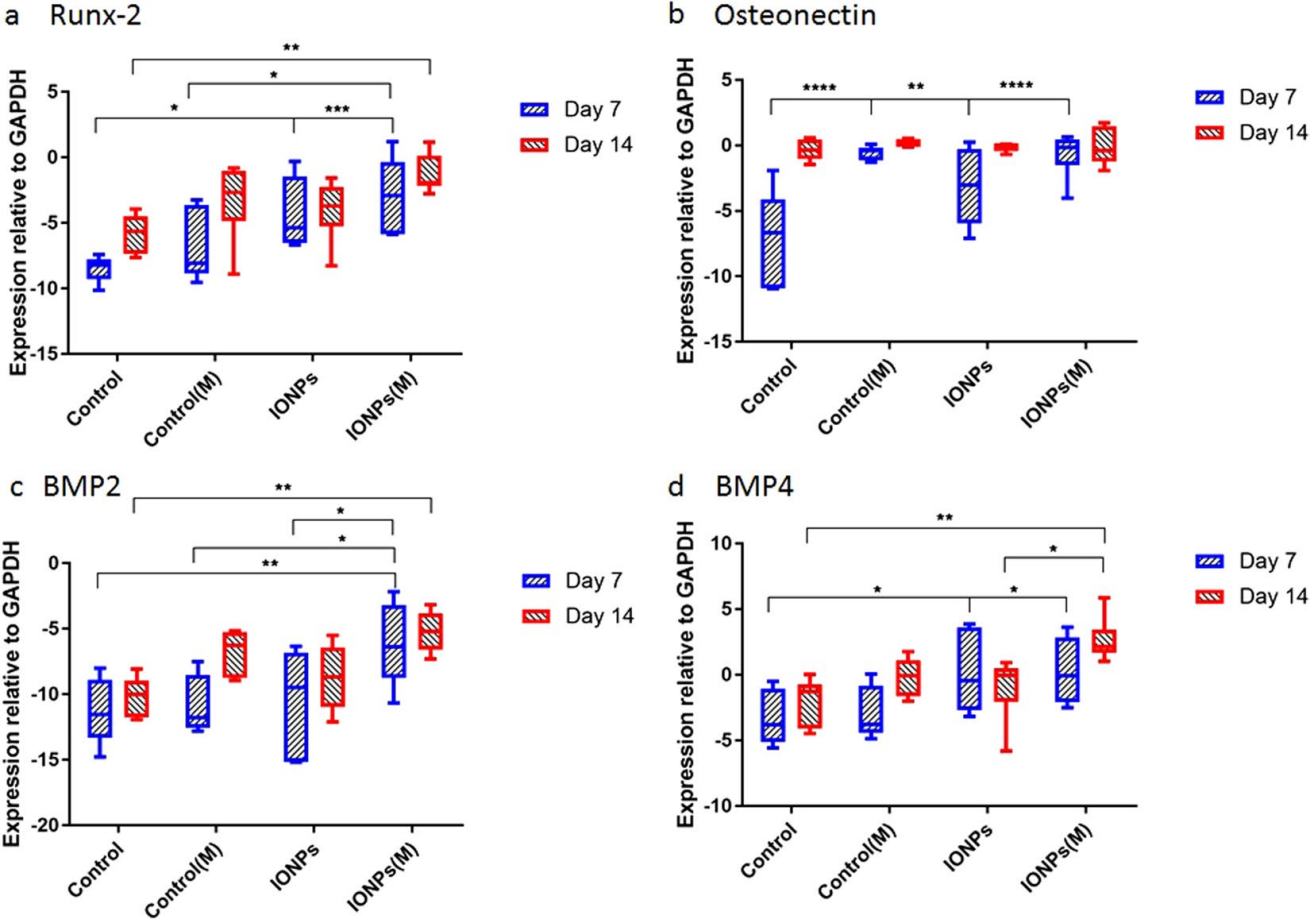
Relative expression of (**a**) Runx2, (**b**) osteonectin, (**c**) BMP-2 and (**d**) BMP-4 normalised to GAPDH after 7 and 14 days of culture (n = 3, *p < 0.05, **p < 0.01, ***p < 0.001, ****p < 0.0001).

To understand the responses of cell-seeded collagen scaffolds to IONPs and SMFs at the molecular level, the expression of Runx2, ON, BMP-2 and BMP-4 were investigated by PCR. The expression of Runx2 normalised to GAPDH is illustrated in Fig. 4a. A 7 day treatment of SMFs alone had no significant effect on the expression of Runx2, whereas increased expression can be found with the combination of SMFs and IONPs. After 14 days, the expression of Runx2 in the control sample increased with increasing culture time. This demonstrates the collagen matrix can mediate the Runx2 expression during osteogenesis. When compared to the controls, continuous treatment with both SMFs and IONPs enhanced the expression of Runx2, whereas this effect cannot be observed with IONPs or SMFs alone. Significant enhancement between the combination condition and SMFs (alone) was observed at 7 days, however, this effect was not continued to 14 days. The expression of ON normalised to GAPDH is illustrated in Fig. 4b. As can be observed, after 7 days, the level of ON expression in the samples treated with IONPs, SMFs and both are higher than that in the control. Particularly, for SMFs and the combination treatment, the level of expressions were the most significant. After 14 days, all samples with different conditions were increased to a similar level, and there were no significant differences observed. This shows that SMFs and IONPs only enhanced the expression of ON within a short time period, and have no significant influence for longer time periods. Figure 4c,d represent the expression of BMP-2 and BMP-4, respectively. When cultured for 7 days, the expression of BMP-2 in collagen, treated with IONPs or SMFs alone, remained at a similar level to the control. However, when treated with both IONPs and SMFs, significant enhancement was observed. Differences between the SMFs (alone), IONPs (alone) and the combination treatment were observed, indicating the stimulating effect of the combination of SMFs and IONPs. After 14 days, the expression of BMP-2 in the control sample did not increase with time, whereas the effect of the combination of IONPs and SMFs on BMP-2 expression was significant. This shows the combination treatment can stimulate the expression of BMP-2 in collagen scaffolds. As for BMP-4, at day 7, the treatment with SMFs did not affect the expression significantly, whereas the incorporation of IONPs, and further exposure to SMFs can up-regulate the expression of BMP-4. When further cultured for 14 days, the effect of IONPs was not significant when compared to the control, as well as the SMFs, whereas a faster stimulation was observed when combining these two together.

## Discussion

The effects of SMFs, collagen matrix and the incorporation of IONPs on the osteogenesis of MG-63 cells have been studied. By exposing to SMFs for 1, 3, 7 and 14 days, stimulating effects of SMFs alone on cell proliferation can be observed for up to 7 days. In the study of Cai *et al*.^41^, increased cell proliferation of MC3T3-E1 cells was observed when exposed under 100 mT SMFs between 4 hours and 7 days of culture. However, cell proliferation decreased afterwards. The reason for not observing an increase in proliferation after 7 days could be that bone responds to dynamic rather than static loadings, and this is related to the peak strain magnitude and loading frequency^10^.

By further introducing IONPs to the SMFs treated collagen model, the stimulating effect can be extended for a longer period (14 days). It has been shown that by introducing IONPs into polymer films under a 1 mT SMFs, the growth of bone cells can be enhanced for up to 15 days^42^. Besides, when incorporating IONPs into polymer nano-fibrous membranes, the MG-63 cell proliferation increased with increasing seeding time and IONPs loading^43^. This can be caused by the interactions between SMFs and IONPs. Magnetic fields induce a high magnetic gradient, which causes displacement of the particles along the gradient vector, when the particles are associated with cells, and this leads to the production of compression and tensile forces on the cell membrane^44^, resulting in a range of cellular responses including changes in intracellular calcium levels. Therefore the biological behaviours of cells can be modified.

Cell differentiation investigations revealed that exposure to SMFs promoted ALP production from MG-63 after 21 days, and the incorporation of IONPs further stimulated ALP production at a later stage (Fig. 3a). ALP is one of the key substances that indicate whether osteoblasts have entered the period of extracellular matrix development and maturation. Previous studies have found that osteoblast-like cells expressed greater ALP levels after SMFs exposure^41,42,45^. Results suggested that the local regulatory factors produced by SMFs treated cells, including collagen Type I, ALP and OP, were greater than those of the untreated ones. The stimulation effect of SMFs on osteoblast differentiation can contribute to the reorientation and distortion of cell membranes and therefore modify the membrane properties, resulting in higher expression of growth factors associated with differentiation hence leading to higher levels of ALP production.

SMFs, and the combination of SMFs and IONPs promoted mineralisation at day 21 when compared to the controls (shown in Fig. 3b). After 6 weeks, all samples reached similar levels of mineralisation, indicating the completion of cell mineralisation, which is an essential process in osteogenesis. The transition from proliferation stage to matrix maturation is suggested by the up regulation of genes associated with matrix development, such as collagen synthesis and ALP activity. Calcium accumulation starts at the matrix development stage and reaches its maximum during the mineralisation stage. *In vitro* studies showed that SMFs (290 mT) combined with osteogenic induction could enhance early extracellular calcium and bone mineralisation of dental pulp cells^46^.

MG-63 cell lines have been used extensively for *in vitro* investigations, however, they have also been reported to have atypical and variable behaviours^47^. Some studies have found that the proliferation rate of MG-63 cells are not very representative of bone cell cultures. This cell line displays rapid cell growth without exhibiting contact inhibition, resulting in cell aggregation and faster proliferation when compared to primary cells *in vitro*^48^. However, in the study of Czekanska *et al*.^38^, they observed that although MG-63 has a much faster proliferation rate when compared to human osteoblasts, the growth kinetics of MG-63 was comparable to human osteoblasts in that the exponential phase was observed between days 2 and 6, followed by the plateau phase from days 6 to 10. In this study, the rapid cell growth enabled us to get consistent results, in comparison with sometime unpredictable primary cell models. Some studies questioned the capability of MG-63 cells to synthesis a correct ECM, as well as ALP production^48^. However, in the study of Andrianarivo *et al*.^49^, they reported that by growing MG-63 cells on type I collagen, an increased induction of alkaline phosphatase activity was observed. Similarly, in the current study, both external SMFs and IONPs stimulated the ALP production of MG-63 cells in collagen, implying that the magnetic field and collagen matrix could play an important role in osteoblastic differentiation and phenotypic expression in MG-63 cells, and potentially for primary cells. The ECM formation is encouraged under magnetic stimulations, this could potentially have a positive influence on primary human osteoblasts.

From the observations in the current study, it was shown that the combination of SMFs and IONPs can induce and stimulate the osteogenesis process, however, the mechanisms remain unclear. Osteogenesis is a complex process mediated by succession of gene activation and expression, therefore an investigation at the molecular level was studied, including the effects on the expression of several key genes, Runx2, ON, BMP-2 and BMP-4. Runx2 was up-regulated by the combination of IONPs and SMFs exposure after 7 to 14 days of culture. Runx2 is the osteoblast-specific product of the Cbfa1 gene, and also a transcription factor that is essential for osteoblast differentiation and bone formation. The expression of Runx2 can also cause a stage dependent increase in the structural and functional proteins, for example, ALP, collagen type I, osteopontin, BSP and osteocalcin in osteoblasts. Runx2 up-regulates the expression of bone matrix protein genes, allowing cells to acquire the osteoblastic phenotype, hence promote *in vitro* and *in vivo* bone formation^50,51^. For example, a study by Tsai *et al*.^52^ demonstrated that, magnetic field induced significant higher expression of Runx2 of mesenchymal stem cells, accompanied with the up-regulation of ALP, collagen type I and OC. Taken together, the results presented in the current study suggest that SMFs exposure may induce an earlier osteogenic induction in MG-63 cell lines by modulating early osteoblastic gene expression of Runx2, hence accelerating the osteogenesis. The expression of osteonectin was up regulated by the addition of IONPs under SMFs after 7 days. However, after 14 days, the level of ON production in the control group reached a similar level to the other treated samples. One possible explanation could be that collagen itself has a significant influence on the expression of ON. ON is a glycoprotein in the bone that binds to both hydroxyapatite and collagen, therefore it plays a vital role in bone mineralisation, cell-matrix interactions, and extracellular matrix regulation. When ON is bound to insoluble type I collagen, the resultant complex binds synthetic apatite crystals and free calcium ions. One study suggested that ON is a tissue specific protein that exhibits several interesting activities, such as linking the bone mineral and collagen phases, perhaps initiating active mineralisation in normal skeletal tissue^53^. The IONPs incorporated model under SMFs was shown to enhance the expression of ON over a 2 week period, which would explain the enhancement in the mineralisation level.

The osteoinductive capacity of BMP-2 and BMP-4 and their critical role as regulators of cell differentiation during fracture repair have been identified^54,55^. A lot of research has been focused on the stimulatory effects of magnetic fields on BMP-2 and BMP-4. Findings from the current study indicate that the expression of BMP-2 and BMP-4 can only be enhanced by the combination of SMFs and IONPs over 14 days of culture. Both BMPs and Runx2 are able to stimulate osteoblast differentiation and bone formation and their interrelationships have also been examined by Gersbach *et al*.^56^. The BMPs provide signals that enhance Runx2 dependent transcription and at the same time, Runx2 provides information necessary for BMPs activity. Therefore, the up-regulation of Runx2 and stimulation of the expression of BMPs, and the enhanced expression of BMPs can further promote the production of Runx2.

The up-regulation of the Runx2, ON, BMP-2 and BMP-4 can be influenced by the modification of cell membrane properties. Bilayer membranes, which are composed of a number of protein and lipid molecules, possess anisotropic diamagnetism in nature^57^. Upon the exposure to SMFs, the phospholipid molecules of the membrane can be rotated by virtue of their collective diamagnetic properties. This can possibly result in over deformation of the cellular membrane, modification of the biological properties of embedded receptors in the membrane and, thereby altering the proliferation kinetics of the cells. Besides, it is generally known that morphological and structural changes to the plasma membrane interfere with many functional and structural features of the cells, leading, for example, to changes in cellular shape, cytoskeleton arrangement, ion flux, receptor distribution^58^. To be more specific, the reorientation of the membrane matrix will influence the embedded ion channels, most likely by producing some degree of deformity of their intra-membranous segment, hence leading to the activation of calcium channels^9^. Therefore, by exposure to external SMFs and the incorporation of IONPs, the cell membrane properties and cell/matrix interactions were influenced, leading to the up-regulation of Runx2, ON, BMP-2 and BMP-4 genes.

## Conclusion

In this study, a bio-mimetic 3D collagen model has been developed, with the ability to incorporate cells and nanoparticles, and can respond to external stimulations. MG-63 cell line and plastic compressed collagen model were successfully used as an *in vitro* model with reproducible and consistent results. In conclusion, SMFs and IONPs together can enhance osteogenesis of MG-63 cells when seeded inside the 3D collagen scaffold, by up-regulating the expression of Runx2, ON, BMP-2 and BMP-4, which is a result of the cell/matrix interactions. The 3D model developed in the study can serve as a superior platform to further investigate biological behaviours *in vitro*, and pave the way for further applications in tissue regeneration and regenerative medicine.

## Methods

### IONPs fabrication

IONPs were prepared by the co-precipitation method^59^. The method involves addition of a base (NaOH or NH_4_OH) to ferric (Fe^3+^) and ferrous (Fe^2+^) chloride solutions under ambient pressure at an elevated temperature. Briefly, 5.41 g of FeCl_3_ $\cdot$ 6 H_2_O (Sigma-Aldrich) were mixed with 1.99 g of FeCl_2_ $\cdot$ 4H_2_O (Sigma-Aldrich), followed by an addition of 400 ml NH_4_OH (Sigma-Aldrich). The resulting solution was left to grow for 30 mins and then washed 7 times by using centrifugation and DI water. The suspension was mixed under sonication (Digital Sonifier 250, Branson Ultrasonics, Danbury, CT, USA) at 40 W in between each wash.

### Formation of collagen gel

Acellular collagen gels were made by titrating the pH of rat tail type I collagen (First Link, Birmingham, UK. 2.10 mg/ml in acetic acid). To make a total volume of 10 ml solution, 8 ml of acid soluble collagen was needed, with the addition of 1 ml 10X DMEM (Gibco, Paisley, UK). The obtained solution was neutralised with 1M NaOH (Sigma-Aldrich), until a colour change from yellow to cirrus pink was observed^18^. The neutralised gel requires the same pH value to ensure a consistency. The remaining 1 ml was made up with DMEM (Sigma-Aldrich) with and without cells. MG-63 cell line (Homo sapiens, osteosarcoma, ATCC) were used in this study. This collagen gel was then set in a 24 well plate with an amount of 1 ml per well, followed by incubation at 37 °C with 95% relative humidity and 5% CO_2_ for 30 minutes.

### Plastic compression

The plastic compression protocol is modified from a study of Brown.*et al*.^18^. Briefly, collagen gels were set in 24-well plate and covered by several layers of filter paper (Whatman grade I) on the top. A cylinder plunger roll (height = 3.7 cm, diameter = 1.5 cm) made by filter paper was laid on the top of each gel for 5 minutes. Extra weights can be added on top of the filter paper roll to accelerate the process, with approximately 15 g per well. The extra liquid component is absorbed by the plunger and leaves a thin layer of collagen gel with a thickness of 75–100 *μ*m. IONPs can be embedded inside the collagen matrix at the point of self-assembly. After the completion of plastic compression, the plunger roll was removed. Cell medium (1 ml) was then added to each well immediately to keep the samples hydrated. The filter paper discs separating the plunger and the hydrogel can then be removed at this point. Samples were then used or cultured as required.

### Setting up exterior magnetic field

The exterior static magnetic fields (SMFs) was designed and simulated by computational software (ANSYS Maxwell) with a magnetic field strength with the range of 72–144 mT (at the centre of the field, with a diameter of 1 cm). Neodymium magnets were used as the sources of permanent magnets. A magnet holder was then designed via ANSYS SpaceClaim and transferred to Magics 19 for selective laser sintering (SLS) (EOSINT P100), with a layer height of 0.1 mm. The material used is biocompatible Nylon (PA 2200).

### Alamar blue assay

The proliferation of MG-63 cell lines was assessed by using alamarBlue (Bio-Rad). Approximately 10^4^ osteoblasts were seeded into the compressed collagen scaffolds incorporated with nanoparticles. Cell seeded scaffolds were left for periods of 1, 3, 7 and 14 days in the incubator (37 °C, with 5% CO_2_). 1 mL of DMEM was added to each well and replaced every two days. At each time point, 100 *μ*l of the reagent was added into each well. The plate was then incubated at 37 °C, with 5% CO_2_ for a period of 2 h to allow the reaction. Afterwards, 100 *μ*l of the supernatant was then removed from relevant wells (n = 3) and added to a 96-well plate (Falcon). Fluorescence was measured using a fluorescent plate reader (FLUstar OPTIMA) at wavelengths of 544 nm (excitation) and 590 nm (emission).

### Alkaline phosphate quantification

Approximately 10^4^ cells were incorporated in 1 ml of collagen gel with various nanoparticles, followed by compression and incubation at 37 °C, with 5% CO_2_. DMEM changes were carried out every two days. After incubation for 7, 14 and 21 days, the scaffolds were removed into a 24-well plate and washed by PBS. Equal amounts (500 *μ*l) of substrate reagent was added to each well. One tablet of 4-nitrophenyl phosphate disodium (SIGMA-A, Dorset, UK) was mixed with 8 mL 0.1 M Trizma Hydrochloride (3.94 g Tris in 500 ml DI water, pH = 9.5) and 15 *μ*L of 2 M MgCl_2_ (9.521 g MgCl_2_ in 50 ml DI water). The 24-well plate was left at room temperature for 15 mins for the reaction to take place. 100 *μ*l of 0.5 M NaOH (10 g NaOH in 500 ml DI water) was then used to stop the reaction. Subsequently, 100 *μ*l was removed from each well (n = 3) and transferred into a 96-well plate and the absorbance read at 405 nm (reference at 670 nm) using a plate reader (FLUstar OPTIMA).

### Alizarin red S staining

The samples were examined after 1, 21 and 42 days of culture. Scaffolds were washed three times with PBS and fixed with 4% paraformaldehyde for routine wax embedding and sectioning. The sectioning process was done by a microtome with a thickness of 5 um each. Sectioned samples were then been collected in hot water and dried for 30 mins. After drying, specimens were de-waxed and cleaned in xyline for 2 mins, followed by several washes of 100% ethanol, 90% ethanol and 70% ethanol. A protocol for staining has been developed for this study. Basically, 1 g ARS dye was dissolved in 50 ml deionized water and the pH was adjusted to 4.1–4.3 with 10% ammonium hydroxide. 1 ml of ARS solution was added on each slide for 30 seconds and DI water was used to wash off the excess dye adsorbed on the scaffold surface. Presence of mineral deposition (red-orange colour) was evaluated using a Nikon eclipse TE2000-5 optical microscope. For the quantification of the level of mineralisation, the stained samples were desorbed with the use of 10% (w/v) cetylpyridinium chloride in 10 mM sodium phosphate (pH 7.0). The dye was then collected and absorbance read at 540 nm using a plate reader (FLUstar OPTIMA).

### Histology

Histology samples were prepared and stained with haemotoxylin and eosin (H & E). Three different sections were cut from each sample and examined with two magnifications (10X and 40X). The cell numbers in each image were counted and plotted with average number and standard deviation (n = 3). Images were analysed by image analysis software (Image J) to quantify cell numbers. Histology specimens were fixed with 4% paraformaldehyde for routine wax embedding and sectioning. The sectioning process was done using a microtome with a thickness of 5 *μ*m. Sectioned samples were then collected in hot water and dried for 30 mins. After drying, specimens were de-waxed and cleaned in xyline for 2 mins, followed by several washes of 100% ethanol, 90% ethanol and 70% ethanol. After air drying, H&E staining was then used for staining the samples for 30 seconds each, followed by rinsing under running water. After staining, specimens were dehydrated by 70% ethanol, 90% ethanol and 100% ethanol and xyline for future storage, with cover slips mounted on top.

### Microstructure examination by TEM

A Philips CM120 Transmission Electron Microscope was used to examine the cellular structure of cell-seeded collagen tissues. Cell seeded collagen scaffolds were stabilised in Karnovsky’s fixative (2.5% glutaraldehyde/2% paraformaldehyde/0.1 M PBS) to preserve structure details, follow by dehydration and then infiltrated with a liquid resin, which is hardened by gentle heat. Resin blocks containing the tissue can therefore be sectioned and stained with heavy metal solutions for observation in the TEM. Firstly, pieces of the fixed tissue were placed in screw cap microtubes with PBS and washed 3 times of 10 mins each. Secondly, the PBS was discarded and the samples were postfixed in 1% osmium tetroxide solution for 1 hour at 4 °C. Then osmium solution was removed with a pipette. Samples was washed 3 × 15 mins with distilled water, followed by dehydration through graded ethanol solutions of 30%, 50%, 70% and 90% with 2 × 15 mins each, and finally in 100% ethanol 3 × 15 min. The infiltration with liquid resin was achieved by infiltrating the tissue in 40% resin for 3 hours, overnight in 60% resin and then 100% resin for 6 hours at room temperature. After final infiltration, tissues were placed in labelled embedding moulds with fresh resin and polymerised in the oven at 70 °C overnight to be examined. The specimens were sectioned into 70 nm and stained in uranyl acetate for 2 hrs, followed by lead citrate for 5 mins.

### RNA extraction

RNeasy Mini Kit (Qiagen 74104 and 74106) was used to extract the RNA from the samples. Firstly, 350 *μ*l Buffer RLT was added to the sample in a micro-centrifuge tube and mixed well to release cells. Samples were stored at −80 °C if not used immediately. Secondly, 250 *μ*l ethanol (96–100%) was added to the diluted RNA and mixed well by pipetting, followed by transferring the samples (700 *μ*l) to an RNeasy Mini spin column placed in a 2 ml collection tube. 350 *μ*l of Buffer RW1 was then added to the RNeasy column. 10 *μ*l DNase I stock solution was added to 70 *μ*l Buffer RDD. This was mixed well by gently inverting the tube. Then the DNase I incubation mix (80 *μ*l) was directly added to the RNeasy column membrane and left for 15 mins at 20–30 °C. Then 350 *μ*l Buffer RW1 was added to the column, followed by 2× of 500 *μ*l Buffer RPE (with the addition of 4 volumes of 96–100% ethanol for a working solution) to wash the membrane. Centrifugation at ≥8000 × g for 15 s was needed in between each step. Finally, the RNeasy spin column was placed in a new 1.5 ml collection tube with an addition of 40 *μ*l RNase-free water directly to the spin column membrane, centrifuged for 1 mins at ≥8000 × g to elute the RNA. The isolated RNA was quantified by a Nanodrop spectrometer.

### Reverse transcription with elimination of genomic DNA for quantitative, real-time PCR

To eliminate the genomic DNA, 2 *μ*l of buffer GE, 50 ng of template RNA with variable RNase-free water were used to make a total volume of 10 *μ*l reaction. The samples were incubated for 5 mins at 42 °C and then placed on ice immediately. Then the reverse transcriptase master mix was prepared by adding 4 *μ*l 5 × buffer BC3, 2 *μ*l of RE3 Reverse Transcriptase Mix and 4 *μ*l of RNase-free water to make a total volume of 10 *μ*l solution. This master mix was incubated at 42 °C for 5 mins. Then the template RNA and reverse transcriptase master were mixed together, followed by incubation at 42 °C for 15 mins and 95 °C for 5 mins. Then 30 *μ*l RNase-free water was added to each reaction and mixed by pipetting up and down several times. The RT SYBR Green Mastermix was prepared as below. 0.5 *μ*l of 10 *μ*mol of forward primer and 0.5 *μ*l of 10 *μ* mol of reverse primers were added together with 4 *μ*l of SYBR Green and 3 *μ*l of RNase-free water to make a 8 *μ*l solution. Finally, 2 *μ*l of the cDNA and 8 *μ*l of the SYBR Green Master Mix was added into one well to make one reaction. The primers used are: GAPDH: 5′ TGCACCACCAACTGCTTAGC 3′ and 5′ GGCATGGACTGTGGTCATGAG 3′^60^; Runx2: CCAACCCACGAATGCACTATC 3′ and 5′ TAGTGAGTGGTGGCGACATAC 3′^61^; Osteonectin: ATTGACGGGTACCTCTCCCA 3′ and 5′ GAAAAAGCGGGTGGTGCAAT 5′; BMP-2: 5′,TTTCAATGGACGTGTCCCCG 3′ and 5′ AGCAGCAACGCTAGAAGACA 3′ and BMP-4: 5′ CGTCCAAGCTATCTCGAGCC 3′ and 5′ CGGAATGGCTCCATAGGTCC 3′ (designed by using Primer-BLAST)^62^. The reaction conditions were as follows: incubation at 95 °C for 2 mins, denaturation at 95 °C for 10 s, annealing at 60 °C for 5 s and polymerisation at 72 °C for 25 s, followed by a final extension at 76 °C for 1 s, for 45 cycles. Quantitative analysis was performed according to the ABI protocol. The threshold cycle (Ct) value was calculated from amplification plots. The dCt value for each sample was obtained by subtracting the Ct values of the housekeeping gene, GAPDH. The data are represented for triplicate readings.

### Statistical analysis

The experiments of biological property evaluation were performed in triplicate. The results are represented as mean ± standard deviation for n = 3. Statistical analysis was carried out by analysis of variance (ANOVA) to determine the presence of any significant differences between groups, and significant level was set at p < 0.05.

## Data Availability

The datasets generated during and/or analysed during the current study are available from the corresponding author on reasonable request.
